# Factors affecting the experience of joined-up, continuous primary care in the absence of relational continuity: an observational study

**DOI:** 10.3399/BJGP.2023.0208

**Published:** 2024-02-06

**Authors:** Patrick Burch, William Whittaker, Peter Bower, Katherine Checkland

**Affiliations:** Centre for Primary Care and Health Services Research, University of Manchester, Manchester.; Manchester Centre for Health Economics, University of Manchester, Manchester.; Centre for Primary Care and Health Services Research, University of Manchester, Manchester.; Centre for Primary Care and Health Services Research, University of Manchester, Manchester.

**Keywords:** continuity of care, coordination of care, general practice, healthcare design, patient experience, primary care

## Abstract

**Background:**

There is an international trend towards the at-scale provision of primary care services, with such services often provided in different settings by a clinician unfamiliar to the patient. It is often assumed that, in the absence of relational continuity, any competent clinician can deliver joined-up, continuous care if they have access to clinical notes.

**Aim:**

To explore the factors that affect the potential for providing joined-up, continuous care in a system where care is delivered away from a patient’s regular practice, by a different organisation and set of staff.

**Design and setting:**

Case studies of two extended-access providers in the north of England.

**Method:**

Case studies were carried out between September 2021 and January 2022 in two sites. Data collected included observations of patient–healthcare professional interactions, interviews with staff and patients, and documentation. Analysis took place using a constant comparison approach. Data were coded. A model of the factors affecting continuity was constructed.

**Results:**

The potential for joined-up, continuous care appears dependent on staff, patient, and system factors. This includes diverse elements such as the attitude of clinicians to care coordination and the ability of an organisation to retain staff.

**Conclusion:**

Healthcare systems increasingly rely on the assumption that any competent clinician can deliver joined-up, continuous care if they have access to clinical notes. This appears not to be the case. This study presents a model of factors affecting the patient’s experience of continuity. The model needs validating in in-hours general practice and other settings.

## Introduction

Continuity of care has long been considered a key element in the delivery of good-quality primary care.^1^ Its precise definition and place in health care and research has been debated, and has subsequently evolved, over the last 40 years.^2^^,^^3^ It can refer simply to a continuous relationship between a patient and a healthcare professional (relational continuity). However, it can also encompass a range of concepts and paradigms, including biomedical, sociotechnical, and health systems factors.^4^ Continuity can be viewed from several perspectives — that of the patient, the clinician, and the health system. This article considers continuity from the perspective of the patient.

There are several conceptual models of patient-experienced continuity.^5^^–^7 The commonly cited Haggerty model defines continuity as: *‘the degree to which a series of discrete healthcare events is experienced as coherent and connected and consistent with the patient’s medical needs and personal context’*.^6^ It then breaks continuity down into three principal components:

- relational continuity — an ongoing relationship between the patient and one (or more than one) provider of health care;
- informational continuity — clinicians and patients having appropriate access to information to enable health care; and
- management continuity — the extent to which the approach to health care over time, and potentially between different providers, is responsive, joined up, and coherent.

It can be claimed that Haggerty’s concept of management continuity is the same as care coordination,^7^ and some have argued that Haggerty’s model could be simplified into just two elements: relational continuity and the seamless delivery of care.^8^ However, the model recognises that there is interplay between the different elements of continuity and that elements of continuity cannot always be neatly categorised. Because of its relative simplicity, flexibility, and widespread use, the current authors use the Haggerty model of continuity as their conceptual lens and it was the basis for this research. To clarify the perceived overlap between ‘coordination’ and ‘management continuity’, the term ‘coordination’ is used in this article to refer to the tasks/organisation of patient care,^9^ and ‘management continuity’ is used to refer to how joined up and coherent that process may appear to the patient.

**Table:** How this fits in

The way that many modern healthcare systems are designed increasingly relies on the assumption that, in the absence of relational continuity, any competent clinician can deliver joined-up, continuous care if they have access to clinical notes. This study of a primary care environment, where patients are usually seen by a clinician they have not met before, demonstrates multiple connected patient, clinician, and system factors that appear important for a patient to experience joined-up, continuous care. Considering these factors in the design of primary care systems may have the potential to improve the experience for patients.

The patient’s experience of continuity is, to a degree, subjective. Tools, such as questionnaires, which attempt to provide quantitative measures of patient-experienced continuity, ask questions that different patients, experiencing the same episode of care, could legitimately answer in different ways.^10^^,^^11^ For that reason, this study refers to the potential for patients to experience joined-up and continuous care.

There is considerable evidence showing an association between relational continuity of care and positive health outcomes.^12^^–^14 There is also some evidence that improving non-relational elements of continuity, such as improving access to information or improving coordination between clinicians, can contribute to improved health outcomes.^15^^,^^16^ However, as there is no agreed definition of what the important non-relational elements of continuity are, or how to measure them, this evidence comes from work in related areas such as care coordination or integrated health systems. It seems self-evident that non-relational factors may contribute to the extent to which care is experienced by patients as joined up and continuous.^17^ Thus, for example, the failure of a clinician to be aware of information received from another provider may generate in a patient a feeling of lack of continuity, even in the context of a long-term therapeutic relationship. It is, therefore, important to understand the factors that come together to create the potential for patients to experience care that feels joined up and continuous, beyond simply seeing the same clinician.

This article uses qualitative data collected as part of two case studies to understand the mechanisms and factors that may influence patient-experienced continuity. Studies using similar methods (interviews and observations) have been used previously to understand complex phenomena such as continuity.^18^^,^^19^ The case studies examine a primary care system in which access is prioritised over continuity. Extended access is an England-wide scheme that enables patients to access routine care in the evenings and at weekends.^20^^,^^21^ Care through extended access is often delivered away from a patient’s regular practice, by a different organisation, and different set of staff. Studying this ‘extreme’ example of a system where continuity will likely be more difficult to achieve enables us to understand the factors that affect the potential for continuity. The data from the case studies are used to create a model of how different elements may interact to affect the potential for patients to experience joined-up and continuous care. The factors that make up this model are then discussed in detail.

## Method

This was a two-site comparative case study of extended-access providers in the north of England (Table 1) conducted between September 2021 and January 2022. Patients using the services were booked appointments by their registered general practice and usually saw a clinician they had not met before. Appointments were a mixture of face-to-face and telephone, and for a mixture of routine and urgent issues. This study was based on the case study methodology described by Stake.^22^ Data collection included observation of 30 hours of patient–healthcare professional interactions with 11 different clinicians (six clinicians in Site A and five in Site B) in seven different extended-access hubs, interviews with staff and patients (24 interviews in Site A and 23 in Site B; of the clinicians observed all six at Site A were interviewed and four of them at Site B were interviewed (Table 2), and organisational documents and protocols. Data were gathered by one researcher, a practising GP and academic. Data analysis and coding were performed by two researchers. All authors contributed to the development of the final conceptual model.

**Table 1.** Details of case study extended-access providers

**Variable**	**Site A**	**Site B**
Number of practices covered	75	37
Number of sites	4	5
IT integration	Variable	Good
Predominant clinical staff	GPs	Advanced nurse practitioners
Default appointment type	Face-to-face	Telephone
Patients clinically triaged before appointment?	Yes	No
Staff able to follow up within the hub?	No	Yes

**Table 2.** Details of interviewees

**Interviewees**	**Site A**	**Site B**
GPs/ANPs	8	4
Administration staff, managerial staff, clinical leads and commissioners	3	7
Local practice GPs/ANPs	1	2
Patients	10	10

*Staff have only been included in one category. However, some staff could be included in more than one category, for example, several hub GPs, ANPs, and receptionists worked in practices that were local to the service; hub clinical leads were also involved in the managerial and clinical aspects of the service. ANP = advanced nurse practitioner.*

Analysis ran concurrently with data gathering. The use of two different sites with contrasting organisational features allowed assumptions and theories generated at one site to be tested on the other. The theory of continuity, as described by Haggerty, was used as a sensitising concept (a lens through which a researcher would initially view data when they have no fixed idea) and in the development of initial codes.^6^ A priori codes were then supplemented with additional codes arising from the data. Analytical memos were written to facilitate the development of themes and higher-level analysis. Emerging findings were discussed regularly at team meetings. Abductive, inductive, and deductive reasoning were used to create a model of factors affecting continuity. These factors were coded and their relationship to one another was examined in existing data. The relationships that emerged from the data guided further data collection and hypothesis testing at both case study sites.

## Results

**Figure 1.**
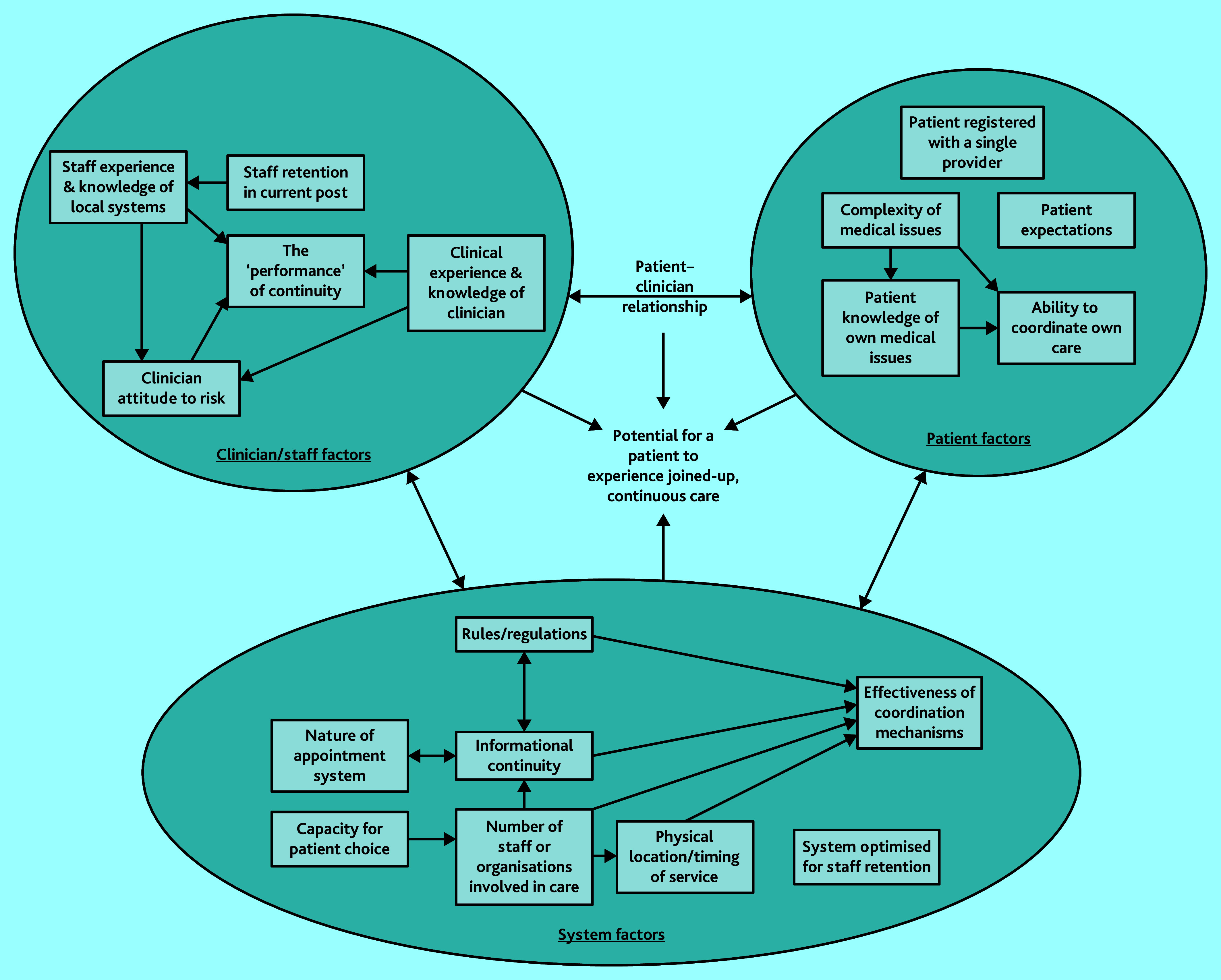
Model of factors influencing patient experienced continuity.

Aspects of care that seemed to be related to generating the potential for an experience of continuity of care were drawn out and charted. These were combined to generate a model of how aspects may interact to generate a patient’s experience of joined-up, continuous care (Figure 1). Subsequent sections draw out, evidence, and explain the model in detail.

### Clinician and staff factors

The staff involved in a patient’s care play a major role in shaping whether a patient experiences joined-up, seamless, and continuous care. In a patient–clinician encounter, the attitudes, knowledge, and experience of the clinician can shape how the care the patient receives links with past and future episodes of care (that is, management continuity). Clinical knowledge is important because the clinician must know how to treat, or facilitate the treatment of, a patient’s condition. However, without knowledge and experience of local health systems, even a medically knowledgeable clinician can fail to provide patients with seamless care. Attitudinal factors also play a role. The clinician’s approach to clinical practice and risk matters: examples include whether they take an entire history again despite it being written down; whether they are willing to take ownership of a patient’s issues; or whether they admit a patient with a borderline case to hospital or monitor them at home. The clinician also has a degree of discretion in how ‘seamless’ they can make a patient’s experience of care, both in perception and in fact. In terms of patient perceptions, the idea that people (including clinicians) behave in different ways depending on who is present and how they perceive their role is referred to as ‘role performance’ in the sociological literature.^23^ The variety of responses from clinicians in situations where a patient could have had more clinician help in coordinating their care shows that the degree to which different clinicians ‘perform continuity’ varies. For example, some clinicians use cues from medical notes to demonstrate to the patient that they know about their previous care, asking questions or making comments designed to indicate knowledge of what has happened before, even if it is not strictly relevant to the current presentation. This has the potential to engender a feeling of continuity for the patient. In terms of actual delivery of coordinated care, clinicians may choose to coordinate an aspect of care themselves, or they may give this task to the patient. Furthermore, non-clinical staff members also play a role in patient care. While clinical knowledge/experience is less important for them, other factors (system knowledge/experience, attitude to practice/risk, willingness to take ownership of a problem, and the option of ‘performing continuity’ either via creating a perception or via their actions) are still likely to affect how staff–patient interactions play out. Finally, the length of time that any given clinician spends working in a particular place will affect many of these things. Staying in one clinical setting will allow the clinician to gain a good knowledge of other local services, as well as making it possible to build personal relationships with patients and with clinicians elsewhere.

The following description illustrates how a clinician at an extended-access provider was able to provide seamless care:

‘*The patient was a lady in her 30s that came from the advanced nurse practitioner (ANP)’s practice. She had irregular vaginal bleeding on the contraceptive implant for several months. The ANP looked through the clinical notes and took a history. She offered her several management options including an injection of Depo-Provera. This is an unlicensed use of the medication. The patient chose to go with this option. The ANP then logged off the hub IT system and, on the same computer, logged onto her practice IT system. Whilst logging on, she told the patient roughly how long she would have to wait before being able to have the injection in the surgery. The ANP loaded up the appointment calendar at her practice and discovered she had no appointments free. She created an additional appointment and booked the patient in. She told the patient she would come in at 08.20 in 3 days’ time, before the start of her booked surgery, to do the injection. She told me that, had the patient been from a different practice, she would have simply asked the patient to contact her own practice to arrange the injection.*’

(Notes from clinical observation of ANP 12, Site A, Case 1)

The ANP in Case 1 had advanced knowledge of gynaecology and was, therefore, able to offer several treatments including an off-licence treatment that not all primary care clinicians might be aware of. She took ownership of the patient’s problem. She had knowledge and experience of local health systems. She went beyond what was required in her role in providing a seamless service for the patient by booking the patient in with herself, a ‘performance of continuity’. Furthermore, as an established staff member present in the local area for a while, she knew that she would be able to follow up the outcome of the chosen treatment, providing an opportunity for a longer-term relationship to develop.

Clinicians had different views as to their role in providing seamless care for patients in the extended-access setting but many felt that the degree of responsibility taken on by the clinician for coordinating care should reflect the ability or vulnerabilities of the patient:

‘[Discussing who should take responsibility for coordinating care between extended-access hubs and general practice] *I think I would do it on an individual basis because I do think that patients have to take onus of their own health and I do want to promote that they’ve got to be proactive and … sort things out for themselves because sometimes patients will … they’ll let you do everything for them. So, I think it’s got to be an individual basis.*’

(ANP 11, Site A)

### Patient factors

As the experience of patient continuity is individual to each patient, patients’ expectations play a key role in shaping their experience. For example, when discussing how it feels when a clinician repeats questions that have already been asked by a different staff member/clinician, different patients reacted very differently to the same experience:

‘*I have to repeat everything. I have to ask them to look at my notes and it’s just a nightmare*.’

(Patient 29, Site A)

‘*A little bit, yeah. You would think, if they could have the information in front of them, they could read up on it. I don’t know if it’s like a standard practice thing, so they know whether you’re lying or not, or putting stuff on, ‘cause you’re having to repeat yourself, I don’t know*.’

(Patient 36, Site B)

‘*I don’t mind that, because even though they’ve got the shared information, they should really be checking things, and things might have changed anyway*.’

(Patient 40, Site B)

Different patients have different expectations as to whether certain tasks of coordination should be undertaken by themselves or by healthcare staff. Physical, mental, social, or psychological factors can also affect the degree to which patients are able or willing to coordinate their own care. In situations where informational continuity of the healthcare record is limited (for example, where clinical staff cannot see the entire record), a patient’s knowledge of their own medical information can help improve their experience of continuity. Several consultations were observed where neither the patient nor the clinician knew why an appointment had been booked. In other consultations, in-depth patient knowledge of medical issues enabled the co-construction of an understanding of the problem and the development of an appropriate management plan, potentially avoiding unnecessary medication and/or repeat investigations. On the other hand, the complexity of a patient’s problems can negatively impact their experience of continuity, and patients who move around frequently are unlikely to experience joined-up care, as records and information must be passed to new care providers.

The interview below illustrates how medical complexity interacts with the design of health systems (and potentially clinician/staff factors) in creating inconvenience and poorly joined-up care. At the opposite end of the spectrum, many patients were observed and interviewed who had less complex, more straightforward health problems, who felt they received appropriate, seamless care:

‘*I have to have a diabetic foot check, and because I’ve got* [other] *problems with my feet, I see a podiatrist occasionally, and I say, “Oh, while I’m here, can you do the diabetic foot check?”; “No.” “Well, why not, I have to have it done every year, so why can’t you do it?”; “Well, it doesn’t say that’s what you’ve come for.” I mean, it takes about a minute, and if it’s not on the computer that that’s what I’ve gone for, they don’t do it, so then I’ve got to try and make an appointment somewhere else and get it done. And it just doesn’t make sense, it’s just a complete waste of money and everybody’s time*.’

(Patient 28, Interview, Site A)

Different patients took different levels of ownership over their health problems and coordinating between services. Some went to considerable lengths to coordinate their care between services, while others assumed that coordination would occur automatically until they experienced otherwise. It varied if patients were informed about who was responsible for coordination of their care.

### System factors

The design of healthcare systems can play a large role in affecting the potential for a patient to experience continuity. The number of staff members and organisations involved in a patient’s care can impact on patient continuity and the ability of staff members to try to provide a seamless service to patients. Moreover, staff turnover is a major factor impacting on the extent to which patient experience of continuous, joined-up care can be delivered. Staff were observed and interviewed who had regularly worked in the same extended-access hub since the inception of the service, while others had moved between hubs or had only recently started working in the service. New staff members do not know systems, and continual turnover can be disruptive. System factors are therefore important in the extent to which they improve staff satisfaction and thereby reduce turnover.

Case 2 illustrates how limited capacity in services limits patient choice and can negatively affect the experience of continuity:

‘*Patient 2 was a lady in her 70s with a rash. She had seen a pharmacist yesterday who advised her that she needed to see a doctor. She had rung her doctors who she said told her there were no appointments and advised her to ring 111. 111 had passed her notes back to the GP saying that she needed to be seen by them within 24 hours. The GP surgery had then made an appointment for her at the extended access hub. The GP examined the rash, diagnosed shingles, and gave her a prescription. She would then need to return to a pharmacist to pick up this medication.*’

(Notes from clinical observation of GP 9, Site B, Case 2)

The physical location, timing, and modality of appointments can also be important. Aside from patients having to travel further or attend at an inconvenient time, these factors can affect the treatment a patient receives. Some patients using extended-access hubs co-located in their registered GP practice did not realise that they had been booked in with a different service. Other patients described difficulties they faced attending an extended-access hub because of the location and/or timing of appointments when they would rather have been seen in their registered practice. Some clinicians reported that they were more likely to admit some patients to hospital when they were seen at particular times because of increased clinical risk or lack of available advice from other clinicians.

The flow of clinical information (informational continuity) is a well-recognised factor that contributes to the potential for patients to experience continuity. Clinicians with access to clinical notes were able to recommend treatment that linked in well with the previous treatment a patient had received. Rules and procedures, although usually well intentioned, can have the effect of impeding the patient’s experience of continuity. Rules and procedures around data protection, for example, can stop clinicians from effectively following up patients or make for some awkward patient encounters, such as that in Case 3:

*‘The ANP called patient 2 — listed in the booking as a man in his 20s. The ANP had not accessed the patient record. He explained to me, he was not allowed to access it until the patient had given explicit consent. The person answering the phone said he was the patient’s father. He explained that his son had autism and couldn’t communicate well himself. It was evident that he expected to have a conversation about his son with the ANP and sounded surprised and put out when the ANP was insistent that the son/patient gave consent for him to speak to the father. After a prolonged wait the patient came to the phone and consented. The ANP then asked for additional consent to gain access to the son’s clinical record*.’ (ANP 101, Site A, Case 3)

The booking systems at both sites were not designed to provide relational continuity. However, clinicians at Site B were able to book patients’ follow-up appointments, and often used this to create short-term relational continuity. Clinicians at Site A were unable to do this and rarely saw the same patient more than once.

Continuity can be improved by having coordination mechanisms in place. However, it is important that these mechanisms are known to staff, easy to use, and effective. Otherwise, staff will circumvent them or ask patients to coordinate their own care. In both case study sites there were several procedures for coordination in place that staff generally did not follow. They explained that this was because they did not believe that tasks would be acted on, were not aware of the procedure, or thought that another method of coordination was more effective. At its worst, such complex and user-unfriendly processes might contribute to increased staff turnover or difficulties in recruiting, as staff may be reluctant to work in a setting in which complex workarounds are required.

### Synthesis: a model of factors impacting patient experience of continuity

The extent to which it is possible for patients to experience continuity of care was found to be complex and mediated by multiple factors. Relational continuity was limited by design of the booking and appointment system. Despite this, some staff managed to provide it in the short term. Informational continuity, in terms of clinicians having access to clinical notes, was generally present. However, the ability for patients to experience continuity was sometimes hampered by a lack of effective coordination between extended-access and in-hours general practice. Whether patients experienced management continuity (joined-up, coherent care) was influenced heavily by the complexity of the patient’s condition, clinicians’ behaviour, and what the patient perceived as joined-up and coherent care. To what degree continuity is something that ‘just happens’ when the appropriate elements are in place and to what degree individuals need to take responsibility for informational continuity, decision making, and coordination to ensure the delivery of continuity, appears to vary between cases, often depending on the complexity of the patient’s health needs.

While longer-term clinician–patient relationships might seem axiomatically to represent ‘continuity’, the results of this study suggest that the complexity of modern medicine means even patients who know the clinician that they are seeing might not experience seamless and continuous care, as many other factors are also important. Moreover, the findings of this study suggest that, even in the absence of those longer-term relationships, an experience of continuity can be delivered by optimising systems and modifying individual behaviours to prioritise this aspect of the patient experience.

## Discussion

### Summary

When patients are seen outside of their usual primary care setting, multiple patient, staff, and system factors interact to create the potential for a patient to experience continuity (or discontinuity) of care. Although the experience of continuity is unique to an individual patient, consideration of these factors when planning healthcare systems could lead to improvements in experienced continuity for patients. Moreover, while longer-term clinician–patient relationships provide an important route to support the delivery of experienced continuity, surrounding system factors are also important.

### Strengths and limitations

To the authors’ knowledge, this is the first study that has used qualitative data to generate a model of how and why patients may experience continuity of care. By examining what happened when patients were seen outside of their normal practice systems, it was possible to identify the factors affecting continuity that may not have been evident in routine general practice. This, along with the large amount of observational and interview data collected, allowed a model to be generated, tested, and refined. However, this model has not been tested outside of the two case study sites. It has not been tested in the context of routine general practice, and this will be important if it is to be useful. Relational continuity plays a major role in the patient’s experience of continuity, but this was rare in the case study sites in the study because of their nature as extended-access hubs. However, individual clinicians were observed who were striving to develop those longer-term relationships, and the model highlights some elements that may enable or inhibit the development of such relationships. While the study authors were reflexive in their data collection and analysis, the fact that one of the authors is a practising clinician may have influenced the practice of some observed clinicians and of interview data that was collected.^24^

### Comparison with existing literature

Relationship-based care, a method of practice promoted by some primary care professional bodies, incorporates the concept that the patient–clinician dynamic is important, even if just for a one-off encounter.^25^ The findings of the current study provide evidence for factors that have the potential to influence this relationship. The degree of responsibility that clinicians feel towards patients and the extent to which they take ownership of patients’ health problems has previously been shown to be related to the length of time they have known the patient.^26^ However, this study suggests that some clinicians feel a sense of ownership even when they see patients for the first time or in a short-term setting. This may be seen as usefully ‘performing’ continuity, or alternatively it may be seen as disempowering patients. Whether this impacts on outcomes requires further research.

Some research has attempted to quantify the patient’s experience of continuity when patients are seen by multiple clinicians, but it has not closely examined the underlying factors that may contribute to the patient’s experience.^10^^,^^11^^,^^27^ The factors that combine to create the patient’s experience of continuity echo findings from research into multimorbidity and treatment burden, in that patients with complex medical issues are not always appropriate for pathways of care, or treatments designed for patients with single diseases.^28^^,^^29^

### Implications for research and practice

Healthcare systems are increasingly complex and fragmented. The growing importance of primary care networks in the UK means that increasingly primary care is being delivered away from a patient’s registered practice, often by a clinician that a patient has not met before. There are multiple initiatives in place to try to improve the patient’s experience of continuity. These include clinicians having access to patient notes, and electronic messaging systems between practices. However, as this study shows, an isolated intervention, however well meaning, is often not enough to enable a patient to experience joined-up, seamless care. In extended-access services, triaging patients so that those patients with more complex issues are dealt with in their registered practice, recruiting staff members who work in local daytime practices, or simplifying coordination mechanisms and rules around data sharing could all potentially improve the patient’s experience of continuity.

Further sociological studies in in-hours general practice, and other settings, using observation and interviews could help validate the model described in this study. Collection of quantitative data about the patient’s experience of continuity and examining their association with patient, clinician, and healthcare system characteristics would be valuable. The model developed in this study potentially provides a framework within which to think about these issues and could, with validation, be used to support quality improvement and work around system design and operation.
